# Early Vascular Alterations in SLE and RA Patients—A Step towards Understanding the Associated Cardiovascular Risk

**DOI:** 10.1371/journal.pone.0044668

**Published:** 2012-09-04

**Authors:** Maria José Santos, Diana Carmona-Fernandes, Helena Canhão, José Canas da Silva, João Eurico Fonseca, Victor Gil

**Affiliations:** 1 Rheumatology Research Unit, Instituto de Medicina Molecular da Faculdade de Medicina de Lisboa, Lisbon, Portugal; 2 Rheumatology Department, Hospital Garcia de Orta, Almada, Portugal; 3 Rheumatology and Metabolic Bone Diseases Department, Hospital de Santa Maria, Lisbon, Portugal; 4 Cardiology Department, Hospital Fernando Fonseca, Amadora, Portugal; University of Southern California, United States of America

## Abstract

Accelerated atherosclerosis represents a major problem in both systemic lupus erythematosus (SLE) and rheumatoid arthritis (RA) patients, and endothelial damage is a key feature of atherogenesis. We aimed to assess early endothelial changes in SLE and RA female patients (127 SLE and 107 RA) without previous CV events. Biomarkers of endothelial cell activation (intercellular adhesion molecule-1 (sICAM-1), vascular cell adhesion molecule-1 (sVCAM-1), thrombomodulin (TM), and tissue factor (TF)) were measured and endothelial function was assessed using peripheral artery tonometry. Reactive hyperemia index (RHI), an indicator of microvascular reactivity, and augmentation index (AIx), a measure of arterial stiffness, were obtained. In addition, traditional CV risk factors, disease activity and medication were determined. Women with SLE displayed higher sICAM-1 and TM and lower TF levels than women with RA (p = 0.001, p<0.001 and p<0.001, respectively). These differences remained significant after controlling for CV risk factors and medication. Serum levels of vascular biomarkers were increased in active disease and a moderate correlation was observed between sVCAM-1 levels and lupus disease activity (rho = 0.246) and between TF levels and RA disease activity (rho = 0.301). Although RHI was similar across the groups, AIx was higher in lupus as compared to RA (p = 0.04). Also in active SLE, a trend towards poorer vasodilation was observed (p = 0.06). In conclusion, women with SLE and RA present with distinct patterns of endothelial cell activation biomarkers not explained by differences in traditional CV risk factors. Early vascular alterations are more pronounced in SLE which is in line with the higher CV risk of these patients.

## Introduction

Chronic systemic inflammation predisposes to accelerated atherosclerosis, a risk that is well known in systemic lupus erythematosus (SLE) and in rheumatoid arthritis (RA) patients [1]. Subclinical vascular lesions develop long before atherosclerosis becomes clinically evident, and they progress more rapidly in SLE [2] and RA [3] than in the general population. Traditional cardiovascular (CV) risk factors do not fully explain this enhanced risk, and the disease itself is considered an independent CV risk factor [1]. In addition, the potential contribution of genetic variants to the development of atherosclerosis in RA patients has been recently highlighted [4], [5]. However, the reported magnitude of the CV risk is several times higher in SLE than in RA [6]–[9], and the reason for this divergence is still incompletely understood.

Endothelial damage is considered the first step in the pathogenesis of atherosclerosis. It correlates with disease progression and predicts CV events in the general population [10]. The importance of endothelial cells (ECs) for vascular health is highlighted by its crucial role in maintaining blood fluidity and in regulating vascular tonus and permeability. Under basal conditions ECs express molecules such as thrombomodulin (TM), which prevent platelet aggregation and the activation of the clotting cascade. Further platelet inhibition is achieved as a result of nitric oxide (NO) synthesis, a major vascular relaxant with anti-inflammatory and anti-proliferative properties. During the inflammatory process, ECs undergo changes characterized by enhanced expression of adhesion molecules, increased transendothelial permeability, and loss of antithrombotic properties [11]. Pro-inflammatory cytokines suppress TM expression and promote its cleavage and release into circulation [12]. In addition, they induce the expression of tissue factor (TF), a procoagulant molecule absent from the surface of the intact ECs [13], shifting the balance towards a prothrombotic state. Furthermore, damaged endothelium loses its ability to produce vasodilators, thus adding to the vascular injury. Endothelial dysfunction is potentially a reversible disorder. Indeed, in patients with active RA, the infusion of infliximab, a chimeric antibody against TNF, has been found to improve biomarkers of endothelial activation [14] and transiently ameliorate endothelial function[15].

In vivo, vascular function can be examined non-invasively by quantifying biomarkers of endothelial activation/damage, by measuring the ability of endothelium to release NO in response to various stimuli or by assessing arterial wall stiffness [16]. Previous data indicate impaired endothelial function both in SLE [17] and in RA patients [18] when compared to non-inflammatory controls. Nevertheless it is unclear whether the magnitude of early vascular changes is similar in these two diseases.

Given the clinical and pathophysiological particularities of SLE and RA, we hypothesize that endothelial function is differently disturbed in these two patient groups, which could explain the different CV risk. Thus, the major aim of our study was to compare endothelial cell function between SLE and RA as assessed by the measurement of soluble vascular biomarkers and by endothelial function testing, taking into account the presence of traditional CV risk factors and systemic inflammation.

## Materials and Methods

### Subjects

Consecutive SLE and RA women fulfilling the ACR classification criteria and free of clinically manifest CV disease were recruited from the rheumatology clinics of Hospital Garcia de Orta, Almada, and Hospital de Santa Maria, Lisbon, between April 2009 and October 2010. A control group of women without systemic inflammatory diseases was also recruited from the local community and evaluated in the same period. Participants were excluded if they were pregnant, breastfeeding, had impaired renal function (defined as serum creatinine >1.5 mg/dl), or had documented ischemic heart disease (previous infarction, revascularization surgery, angina, or heart failure), cerebrovascular disease (stroke or transient ischemic attack) or symptomatic peripheral artery disease. The study was approved by the Ethics Committee of both hospitals and was conducted in accordance with the principles stated in the Declaration of Helsinki. All participants gave written informed consent.

### Clinical assessment

Demographic data, disease characteristics, current medication, and CV risk profile including blood pressure, serum lipids, fasting glycemia, smoking habits, and body mass index (BMI) were obtained. Patients were diagnosed with hypertension if the measured blood pressure was repeatedly ≥140/90 mm/Hg or if they used antihypertensive medication. The diagnosis of diabetes was made if fasting glucose level was ≥126 mg/dl, or if patients were under pharmacological treatment. Participants were classified as obese if BMI was ≥30 Kg/m^2^. Disease activity was evaluated using the SLE Disease Activity Index 2000 (SLEDAI 2K), [19] and in RA patients 28 joints were examined for tenderness and swelling, and the 4 variable disease activity score (DAS28) was calculated using erythrocyte sedimentation rate [20]. Disease activity was stratified according to the cutoffs of each instrument [20], [21]: remission (SLEDAI 2K = 0 for SLE or DAS28<2.6 for RA patients), low disease activity (≥1 SLEDAI 2K<4, in the case of SLE, or ≥2.6 DAS28≤3.2, in the case of RA), and active disease (SLEDAI 2K≥4 or DAS28>3.2, for SLE and RA patients, respectively).

Fasting blood samples were obtained before any other procedures for measurement of glucose, uric acid, lipids (total cholesterol, high density lipoprotein (HDL) cholesterol, low density lipoprotein (LDL) cholesterol, and triglycerides), inflammatory mediators (C-reactive protein (CRP) and fibrinogen) and soluble vascular biomarkers (sICAM-1, sVCAM-1, TM, and TF).

### Quantification of soluble vascular biomarkers and cytokines

Measurements were performed using commercial enzyme-linked immunosorbent assay (ELISA) based methods according to the manufacturers' instructions. The Human sICAM-1 FlowCytomix Simplex Kit and the Human sVCAM-1 FlowCytomix Simplex Kit (Bender MedSystems GmbH, Vienna, Austria) were used for quantification of adhesion molecules, both using the FlowCytomix ^TM^ Technology. Serum levels of TM were measured using the Human Thrombomodulin ELISA Kit (Cell Sciences ®, Canton, MA, USA) and serum levels of TF were quantified using the AssayMax Human Tissue Factor ELISA kit (Assaypro, St Charles, Mo, USA).

### Endothelial function tests

Endothelial function was assessed by peripheral artery tonometry (PAT). PAT is a noninvasive operator-independent method that evaluates changes in pulse wave amplitude before and after reactive hyperemia. The inter-day variability of this technique in our department is 11% (data not published). The exam was performed using the EndoPAT 2000 device (Itamar Medical Ltd, Cesarea, Israel) as described elsewhere [22] and by assessors blinded to the clinical diagnosis. Briefly, patients were placed in a quiet room, in supine position, with a specially designed finger probe on the index finger of each hand, and a pressure cuff placed on one arm. Patients were recommended to refrain from smoking and drinking coffee or tea during the previous 24 hours and not to eat for at least 6 hours preceding the exam. PAT was continuously measured during a 10-minute baseline period, for 5 minutes after the pressure cuff was inflated to suprasystolic pressure and for 10 additional minutes following the release of upper arm occlusion. Pressure changes reflecting pulse amplitude were transmitted to a computer and reactive hyperemia index (RHI) was calculated as the ratio of PAT signal amplitude after cuff deflation divided by the amplitude of baseline signal, adjusted for fluctuations in the magnitude of the signal in the contralateral finger [22]. Augmentation index (AIx) was calculated from the mean PAT waveform of the baseline period dividing the amplitude of the second systolic peak by the difference between the second and the first peak.

### Statistical analysis

Continuous variables are expressed as means with standard deviations and categorical variables as the number of affected individuals and proportion of the total. Bivariate comparisons of SLE and RA patients were made using Student T-tests, Mann-Whitney, Kruskal-Wallis or χ^2^ tests, as appropriate.

The levels of vascular biomarkers, as well as RHI and AIx, were compared between SLE and RA patients first as crude means using the Mann Whitney test, followed by analysis of covariance (ANCOVA) to adjust for significant and clinically relevant baseline covariates. Likewise, in order to assess the effect of disease activity on vascular biomarkers, RHI and AIx, comparisons between remission and active disease were performed.

Correlation between disease activity and endothelial cell function was studied separately in SLE and RA using Spearman correlation coefficient and partial correlations to control for age, disease duration, cardiovascular risk factors and medication.

Statistical analysis was performed assuming a 5% significance level and using SPSS 17 for Windows.

## Results

In total 127 women with SLE and 107 with RA, were included in the study. A control group of 124 women, mean age 46.9±13.7 years, 98% Caucasian, and 52% postmenopausal was also evaluated as reference. Demographic and clinical characteristics of SLE and RA patients are shown in Table 1. SLE women were younger and had shorter disease duration (8.4±6.5 years) compared to women with RA (10.7±7.3 years, p = 0.01). All lupus patients were ANA positive and 89% of RA patients were positive either for IgM RF or for anti-citrullinated protein antibodies. The use of antimalarials and aspirin was more common in lupus, while more RA patients received methotrexate. Serum concentration of fibrinogen was higher in SLE (SLE 326±147 mg/dl vs RA 276±101 mg/dl; p = 0.01), but CRP levels were similar in both groups (SLE 1.16±3.2 mg/dl vs 1.06±2.5 mg/dl; p = 0.75).

**Table 1:** Demographic and clinical characteristics of SLE and RA women.

	SLE (n = 127)	RA (n = 107)		p value
**Demographic data**				
Age, years	43.9 (13.9)		50.2 (14.1)	0.01
Education, years	8.9 (4.8)		8.3 (5.2	ns
Caucasians, n (%)	110 (87)		95 (89)	ns
Menopause, n (%)	60 (47)		62 (58)	ns
**Traditional CV risk factors**				
Current smoker, n (%)	18 (14)		17 (16)	ns
Hypertension, n (%)	51 (40)		37 (35)	ns
Total cholesterol, mg/dl	189.3 (42.8)		204.0 (33.5)	0.005
HDL cholesterol, mg/dl	56.6 (16.1)		63.9 (18.5)	0.002
LDL cholesterol, mg/dl	113.9 (36.6)		123.4 (28.5)	0.04
Triglycerides, mg/dl	125.7 (88.2)		103.1 (42.8)	0.01
Diabetes, n (%)	9 (7)		5 (5)	ns
Obesity, n (%)	33 (26)		33 (31)	ns
**Medication**				
Antihypertensive, n (%)	51 (40)		32 (30)	ns
Lipid lowering, n (%)	31 (24)		17 (16)	ns
Aspirin, n (%)	28 (22)		6 (6)	<0.001
Hydroxychloroquine, n (%)	97 (74)		20 (19)	<0.001
Methotrexate, n (%)	12 (10)		85 (82)	<0.001
Prednisolone, mg/day	7.8 (10.9)		3.2 (3.5)	0.006

Results are presented as means (SD) or number of affected individuals and (%).

SLE – systemic lupus erythematosus; RA – rheumatoid arthritis; CV- cardiovascular; HDL – high density lipoprotein; LDL – low density lipoprotein, ns – non significant.

### Vascular biomarkers and endothelial function as assessed by PAT

**Figure 1:**
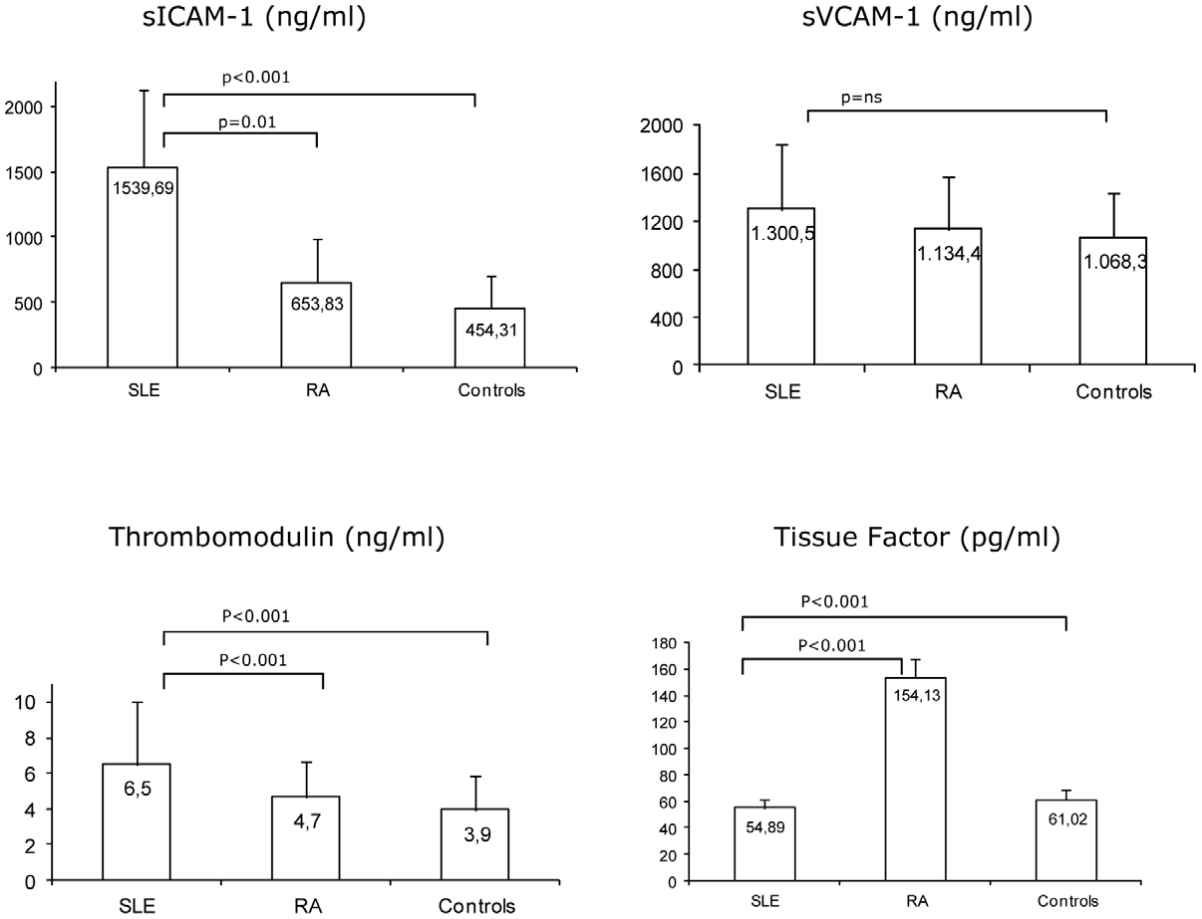
Serum concentrations of vascular biomarkers in SLE and RA patients and non-inflammatory controls. sICAM-1– soluble intercellular adhesion molecule; sVCAM-1 – soluble vascular cell adhesion molecule; RHI–reactive hyperemia index; Aix – augmentation index; SLE – systemic lupus erythematosus; RA – rheumatoid arthritis.

A distinct pattern of soluble ECs biomarkers was identified in SLE and in RA. While sICAM-1 and TM levels were significantly higher, TF was lower in lupus than in RA patients (Figure 1). Differences in sICAM-1, TM and TF remained significant after adjustment for covariates (Table 2).

**Table 2:** Vascular biomarkers and results of PAT assessment in SLE and RA, after controlling for baseline covariates.

	Age adjusted			Adjusted for CV risk factors, disease duration and medication*					
	SLE (n = 127)	RA (n = 107)	p value	SLE(n = 127)		RA(n = 107)		p value	
**sICAM ng/ml**	1436 (600)	778 (651)	0.01	1994(879)	398(898)		0.05		
**sVCAM,** ng/ml	1313 (114)	1129 (125)	0.29	1330 (159)	1160(162)		0.53		
**TM,** ng/ml	6.59 (0.27)	4.54 (0.29)	<0.001	6.47 (0.36)	4.58(0.37)		0.003		
**TF,** pg/ml	56.3 (10.1)	152.6 (10.9)	<0.001	50.1 14.4)	157(14.7)		<0.001		
**RHI** §	2.128 (0.08)	2.203 (0.09)	0.53	2.008(0.11)	2.309(0.12)		0.12		
**AIx** § **, %**	17.9 (1.9)	9.5 (2)	0.003	20.3 (2.5)	8.3 (2.7)		0.007		

Results are presented as estimated marginal means (SE).

* Adjusted for the following covariates: age, disease duration, total cholesterol, HDL, LDL, triglycerides, aspirin, hydroxychloroquine, methotrexate use, and prednisolone dose.

§ RHI and AIx results refer to 87 women with SLE and 75 with RA.

sICAM-1 – soluble intercellular adhesion molecule; sVCAM-1 – soluble vascular cell adhesion molecule; TM – thrombomodulin; TF – tissue factor; RHI – reactive hyperemia index; AIx – augmentation index.

Reactive hyperemia was similar in SLE (RHI = 2.135±0.686), in RA (RHI = 2.194±0.810) and in the control population (2.090±0.579), while AIx was significantly higher in SLE as compared to RA (16% vs 11%; p = 0.04), indicating increased arterial wall stiffness in these patients. This increase remained statistically significant after controlling for differences in baseline characteristics (Table 2).

### Disease activity and endothelial function

Patients presented a broad range of disease activity. The mean SLEDAI 2K was 3.46±4.5 (range 0 to 21) and the mean DAS28 was 4.19±1.4 (range 1.70 to 7.54). Disease was in remission in 41% of SLE and in 17% of RA cases. 39% of SLE patients presented moderate/high active disease defined as a SLEDAI 2K≥4, and 72% of RA had moderate/high active disease according to the DAS28 definition. Except for prednisolone dosage, which was significantly higher in active SLE and active RA than in remission, demographic characteristics, CV risk profile and medication was comparable in quiescent and active disease.

Overall, serum levels of vascular biomarkers were elevated when disease was active, being statistically significant the differences in sICAM-1, TM and TF levels between active and quiescent SLE and in sICAM-1 and TF levels between active RA and remission (Table 3).

**Table 3:** Vascular biomarkers and endothelial function in active disease and in remission.

	SLE (N = 127)			RA (N = 107)		
	Remission	Active	p	Remission	Active	P
**sICAM-1**	713.9 (147)	1952 (100)	0.04	527 (10.2)	668 (4.7)	0.01
**sVCAM-1**	1022 (69)	1419 (47)	0.02	1013 (94)	1171 (44)	0.15
**TM**	6.1 (0.5)	7.2 (0.4)	0.04	4.59 (0.2)	5.01 (0.4)	0.42
**TF**	54.1 (7.3)	58.6 (1.9)	0.08	126.6 (33)	160.4 (15)	0.03
**RHI** *	2.249 (0.13)	1.806 (0.16)	0.06	2.444 (0.21)	2.133 (0.10)	0.20
**AIx** *	14.4 (3.6)	18.5 (2.5)	0.03	12.2 (6.4)	10.8 (2.4)	0.79

Results are expressed as estimated marginal means (SE) adjusted for prednisolone dose.

* RHI and AIx results refer to 87 women with SLE and 75 with RA.

SLE – systemic lupus erythematosus; RA – rheumatoid arthritis; sICAM-1 – soluble inter-cellular adhesion molecule; sVCAM-1 – soluble vascular cell adhesion molecule; TM – thrombomodulin; TF – tissue factor; RHI – reactive hyperemia index; AIx – augmentation index.

sVCAM-1 showed a significant Spearman and partial correlation with lupus disease activity measured by the SLEDAI (rho 0.246 and 0.361; p = 0.007 and p<0.001 respectively) and TM levels correlated with DAS28 (rho 0.301 and 0.250; p = 0.002 and p = 0.005). In SLE patients there was also a significant correlation between sVCAM-1, TM, TF and ESR (rho 0.246, 0.323 and 0.263; p = 0.01, p = 0.001 and p = 0.01, respectively) and between serum TM levels and CRP (rho 0.315; p = 0.001). No significant correlation was found in RA patients between ESR or CRP and vascular biomarkers.

A trend toward lower RHI was observed in active SLE (1.806±0.16) as compared with remission (2.249±0.13; p = 0.06), but no significant correlation was observed between RHI, AIx and SLEDAI or DAS28.

## Discussion

In this comparative study we found distinct patterns of soluble vascular biomarkers in SLE and in RA female patients free from clinically evident CV disease. Lupus patients presented higher serum sICAM-1 and TM levels, while TF was elevated in RA patients. These findings are relevant for understanding the pathophysiology of the increased CV risk in SLE and RA patients, as cell adhesion molecules may represent a link between inflammation and atherosclerosis. In fact, not only are VCAM-1 and ICAM-1 highly expressed on the endothelium overlaying atherosclerotic lesions [23], [24], but an increased serum concentration of these molecules is also related to CV risk factors [25] and incident myocardial infarction [26]. In particular, high serum levels of ICAM-1 represent an independent risk factor for atherosclerosis and a predictor of future CV events [26], [27]. In addition, we observed significantly increased levels of vascular biomarkers in active disease. These observations are in line with previous studies demonstrating that inflammatory mediators, including TNF, IL-6, interferon-gamma (INFγ) [28], IL-18 [29], but also MCP-1 and MIF [30], upregulate endothelial cell adhesion molecule expression. The fact that SLE patients exhibit higher sICAM-1 and also higher fibrinogen concentrations may be relevant in the initiation and progression of atherosclerosis. Indeed, ICAM-1 serves as a binding site for fibrinogen and promotes adhesion and transendothelial migration of leukocytes [31], an important early step in inflammatory vascular disease. We did not find any difference in VCAM-1 serum levels among the studied groups. In animal models, VCAM-1 expression is considered a major early event in the atherosclerotic process [32], and increased sVCAM-1 levels have been reported in lupus nephritis [33]. However, in RA and SLE patients without renal or vascular disease, serum concentrations of VCAM-1 are similar to the control population and the relationship to atherosclerosis is uncertain [34], [35]. Nevertheless, very recently sVCAM-1 was identified as an independent predictor of overall and cardiovascular mortality in SLE[36].

There is growing evidence supporting the relationship between inflammation and thrombotic complications of atherosclerosis (atherothrombosis). Interestingly, TM expression, a molecule with anti-coagulant properties, is reduced during the inflammatory process [12], and increased soluble TM levels probably indicate EC injury. Together with increased TF, which is an initiator of the extrinsic coagulation cascade, this environment may raise the thrombogenic activity of plasma and contribute to cardiovascular events. Higher levels of TF in RA patients as compared to SLE patients might be explained by the contribution of TNF to its expression [37]. Nevertheless, serum levels of adhesion molecules, TM, and TF may not accurately translate endothelial functional expression of these molecules, which is a limitation of our work.

A further effect of proinflammatory cytokines on EC is the inhibition of NO synthesis leading to endothelial dysfunction. In the general population, impaired endothelial function is a critical early step in the development of atherosclerosis [38] and predicts the progression of structural arterial disease independently of conventional CV risk factors [39], [40]. However, studies of endothelial function in inflammatory rheumatic diseases depicted contradictory results [41]–[43], and the relevance of endothelial dysfunction for the progression of atherosclerosis in rheumatic diseases remains uncertain [44]–[46]. Similarly, the improvement following anti-rheumatic medication is not universally supported by the available literature [18]. Using PAT, we did not find any significant differences in RHI neither between patients and controls, nor between SLE and RA. RHI quantifies changes in pulse wave amplitude in response to reactive hyperemia, a measure of microvascular function. In the general population RHI is an independent predictor of adverse cardiac events [47], but its predictive value in rheumatic diseases has not been established. The fact that we have included only females without previous CV events and normal renal function (relatively low risk population) may in part account for the comparable RHI found in patients and controls. In fact, only in more active SLE cases did RHI show a reduction. The follow up of these patients will allow us to ascertain the predictive value of RHI measured by PAT for the development of CV event in SLE and RA patients.

Lupus patients presented higher AIx than RA patients and this difference remained significant after controlling for covariates. In apparently healthy subjects arterial stiffness is an independent predictor of coronary heart disease and stroke [48], but the predictive value of AIx in rheumatic diseases is unknown. Cardiovascular risk factors and disease related features contribute to arterial stiffening in SLE [49] and RA [50]. Shang et al found a correlation between carotid AIx and SLEDAI [51]. Increased arterial stiffness was also associated with RA disease activity in some, but not all, studies [18]. Increased AIx in SLE women probably indicates a worse vascular condition.

Taken together, our observations add to the evidence that the pathogenesis of atherosclerosis associated with inflammation may differ in SLE and RA. Additionally, we found more pronounced early vascular changes in lupus patients, and when the disease is active, which is in line with the higher risk for CV events documented in these patients.
